# Sulfasalazine synergistically enhances the inhibitory effects of imatinib against hepatocellular carcinoma (HCC) cells by targeting NFκB, BCR/ABL, and PI3K/AKT signaling pathway‐related proteins

**DOI:** 10.1002/2211-5463.13052

**Published:** 2021-02-20

**Authors:** Marium M. Shamaa

**Affiliations:** ^1^ Clinical and Biological Sciences (Biochemistry and Molecular Biology) Department, College of Pharmacy Arab Academy for Science, Technology and Maritime Transport Alexandria Egypt

**Keywords:** AKT pathway, hepatocellular carcinoma, imatinib, NFκB, PI3K, sulfasalazine

## Abstract

Hepatocellular carcinoma (HCC) is the third leading cause of cancer‐related fatalities worldwide. Identification of second‐line therapies for patients with progressive HCC is urgently required as the use of sorafenib and/or regorafenib remains unsatisfactory. Imatinib, a small‐molecule kinase inhibitor, is used to treat certain types of cancer, and nuclear factor κB (NFκB) is a positive regulator of cancer cell expansion. The combined use of tyrosine kinase and NFκB inhibitors may have potential for treating HCC. The aim of this work was to assess the potential anticarcinogenic effects of imatinib and sulfasalazine alone or in combination on the human HCC cell lines HEPG2 and Huh‐7. Both drugs were shown to affect the phosphoinositide 3‐kinase/protein kinase B, phosphorylated signal transducer and activator of translation (p‐STAT‐3), breakpoint cluster region protein/Abelson proto‐oncogene and NFκB pathways. At the transcriptional level, imatinib and sulfasalazine were found to synergistically down‐regulate *c‐MET* gene expression. When compared with the activities of either medication alone, combined use of imatinib and sulfasalazine enhanced inhibition of HCC cell proliferation and extended induction of apoptosis. In summary, the presented data suggest that sulfasalazine synergistically potentiates the antitumor effects of imatinib.

## Graphical abstract

The author investigated the potential anticarcinogenic effects of imatinib and sulfasalazine alone or in combination on HEPG2 and Huh‐7 cell lines. Combined use of both drugs inhibited cell growth and promoted apoptosis via the phosphoinositide 3‐kinase/protein kinase B, phosphorylated signal transducer and activator of translation 3 (p‐STAT‐3), breakpoint cluster region/Abelson proto‐oncogene and nuclear factor κB pathways. These results suggest that sulfasalazine synergistically potentiates the antitumor effect of imatinib.

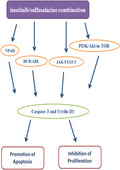

**Abbreviations**
*A*: absorbance
Akt: protein kinase B
BCR: breakpoint cluster region protein
CI: combination index
HCC: hepatocellular carcinoma
MTT: 3‐(4,5‐dimethylthiazol‐2‐yl)‐2,5‐diphenyl tetrazolium bromide
NFκB: nuclear factor κB
p: phosphorylated
RQ: relative quantification
SE: standard error
SSZ: sulfasalazine
STAT: sign transducer and activator of translation protein

Hepatocellular carcinoma (HCC) is well known as the sixth most common harmful cancer type and the third leading cause of cancer‐related fatalities worldwide [1, 2]. No systemic medications for patients with HCC whose illness advances amid sorafenib treatment exist [3]. HCC impacts approximately 1 000 000 people every year worldwide [4]. The most noteworthy HCC predominance worldwide has been reported in Egypt [5, 6]. Sorafenib, a multityrosine kinase inhibitor, is considered to be the only systemic treatment for patients who are not candidates for a locoregional cure [5, 7].

Ongoing studies that address hepatocarcinogenesis have defined critical components of a few signaling pathways within the upgrading of cancer advancement, metastasis and angiogenesis [8]. Several of these pathways, such as the protein kinase B (Akt)/mammalian target of rapamycin, signal transducer and activator of translation (STAT) and the nuclear factor κB (NFκB) complex pathways, play critical roles in HCC advancement [9, 10, 11]. A powerful inhibitor of breakpoint cluster region protein/Abelson proto‐oncogene (BCR/ABL) tyrosine kinase, imatinib, is believed to be the standard of care in HCC control. Imatinib cure resistance frequently occurs because of changes within the BCR/ABL kinase domain [12, 13, 14, 15].

BCR/ABL kinase is considered to be an oncogene that controls and mediates expression of important downstream proteins that have a role in HCC pathogenesis [16]. Also, BCR/ABL was found to be overexpressed in HCC cases [17].

Patients with HCC need early diagnosis and effective therapy such as a second‐generation tyrosine kinase inhibitor. Hence it is vital to recognize variables that may cause patients to have an insufficient reaction to treatment [12]. Imatinib in combination with other agents is presently advised. Many anticancer agents may impact several signaling pathways and lead to much more effective removal of liver cancer cells [18, 19]. The first drug approved for ulcerative colitis was sulfasalazine. It acts as a potent and specific inhibitor of NFκB. Recently, it was suggested that sulfasalazine has anticancer activities against a number of human cancers [20].

The determination of second‐line therapies for patients with progressive HCC is urgent because its improvement after the use of sorafenib and/or regorafenib is still unsatisfactory. This work aimed to assess the beneficial anticarcinogenic effects of the combination of imatinib and sulfasalazine on HCC cell lines.

## Materials and methods

### Chemicals/Reagents

Imatinib mesylate (#STI571) and sulfasalazine (#1576) were purchased from Selleck (Selleck Chem, Houston, TX, USA). 3‐(4,5‐Dimethylthiazol‐2‐yl)‐2,5‐diphenyl tetrazolium bromide (MTT) reagent was purchased from Sigma‐Aldrich (Hamburg, Germany). ELISA kits for human vascular endothelial growth factor (VEGF), phosphorylated (p)‐Akt, active caspase‐3, p‐NFκB, p‐STAT‐3 and p‐BCR/ABL detection were purchased from Eagle Biosciences Inc. (Amherst, MA, USA), Abnova Biotechnology Co. (Cambridge, UK), Quantikine (Minneapolis, MN, USA), RayBio® Biotechnology (Peachtree Corners, GA, USA), Acris OriGene EU (Herford, Germany) and Sigma‐Aldrich, respectively.

### Cell culture

The *Homo sapiens* HCC cell lines Huh‐7 and HEPG2 were obtained from ATCC (Cat: ATCC® HB‐8065™ and ATCC® PTA‐4583; Baltimore, MD, USA). HEPG2 and Huh‐7 cells were preserved in T‐25 flasks at 37 °C and 5% CO_2_ in an adjusted mixture of Dulbecco’s modified Eagle’s medium from Lonza BioWhittaker™ (Verviers, Belgium) and 10% (v/v) FBS from Sigma‐Aldrich [21].

At concentrations of 100 μg·mL^−1^ and 100 U·mL^−1^, penicillin/streptomycin (Lonza BioWhittaker™) was used. PBS, pH 7.2, was obtained from Lonza BioWhittaker™, and 2.5% trypsin was obtained from Gibco™ Life Technologies Corporation (New York, NY, USA).

### Cell viability assay

The MTT test was applied to check cell viability. For the most part, 5 × 10^3^ cells were permitted to grow in 96‐well plates. Imatinib, sulfasalazine and a combination of the two were incubated for 48 h, and then to assist incubation, I added 10 μL MTT solution (0.5%) to the medium for 4 h. The tested concentrations for imatinib included 40, 20, 10, 5, 2.5, 1.3, 0.6 and 0.3 μm. For sulfasalazine, concentrations to 400, 200, 100, 50, 25, 12.5, 6.3 and 3.1 μm were used, and for combination treatment, half of the concentrations of each drug described earlier was used. Half of the concentrations of individual drugs used in the combination group was used with the aim of investigating the possibility of minimizing drug doses when using a combination with the objective of reducing the doses, reducing their toxicities and side effects and, as a result, improving treatment outcomes.

To solubilize the insoluble formazan crystals, I added 100 μL DMSO to each well after removing the medium. The colored complex absorbance (*A*) was determined at 570 nm with a spectrophotometer. All tests were performed in triplicate.

The combination index (CI) was calculated using the following formula:

CI = CdrugAX = AX/CdrugA + CdrugBX/CdrugB

in which drugs AX and BX are the concentrations of drugs used in combination treatment, whereas drugs A and B are concentrations of each drug alone.

### ELISA

VEGF, p‐STAT‐3, p‐NFκB, p‐Akt, p‐BCR/ABL and activated caspase‐3 protein levels in cell supernatants and cell lysate were identified by human ELISA kits following the manufacturer’s instructions. After HEPG2 and Huh‐7 cell cultivation for 48 h, the medium was then centrifuged at 17 741 ***g*** for 5 min. Complete media with 10% FBS were used as a control.

The levels of the previous parameters assayed by ELISA in the samples were determined using linear regression equations. The resulting values were divided by the amount of protein (mg) in the sample to express values relevant to milligrams cellular protein to be normalized to the total protein level. The Bradford method was applied to measure the overall protein level by following the manufacturer's instructions. The experiments were performed in quadruplicate.

### Real‐time PCR

Real‐time RT‐PCR was used to measure the *c‐MET* gene expression level. In brief, total RNA was extracted by using an RNA‐spin™ total RNA extraction kit using silica‐gel membrane adsorption (Jena Bioscience Incorporated, Jena, Germany). A NanoDrop 2000 spectrophotometer (Thermo Fisher, Bedford, MA, USA) was used to measure the concentration and purity of the extracted total RNA.

The *A* of total RNA was measured at 260 and 280 nm using Tris‐EDTA buffer as a blank. The ratio of *A*_260_/*A*_280_ was applied to determine the purity of total RNA. The extracted RNA concentration was expressed as nanograms of total RNA per microliter.

Real‐time RT‐PCR assays were performed using the SensiFast™ One Step RT‐PCR kit with SYBR^®^ Green Hi ROX (#BIO‐73001; Bioline Life Science Company, Swedesboro, NJ, USA), which was designed for highly reproducible first‐strand cDNA synthesis and subsequent quantitative PCR in a single tube, and the internal control used was *β‐actin*.

Relative quantification (RQ) values for the tested gene were calculated using the ΔΔCT method with adjustment for β‐*actin* expression relative to the expression level of control, for which RQ = 1. Log_10_ RQ was applied to calculate fold changes using the formula Log_10_RQ  =  Log_10_ $2^{‐\Delta \Delta C_{t}}$ (Log_10_RQ  = 0 indicates no change in expression; Log_10_RQ = 1 indicates that the tested gene is expressed at a level 10 times greater than that of the control sample; and Log_10_RQ = −1 indicates that the tested gene is expressed at a level 10 times lower than that of the control sample).

The $2^{‐\Delta \Delta C_{t}}$ method was used to calculate the relative expression levels. The sequences of the primers were blasted against NCBI/Primer Blast. The experiments were performed in quadruplicate.

The *c‐MET* and β‐*actin* primer sequences are: *c‐MET* forward, 5′‐GAAAATTGACTTAGCCAACCGAGAG‐3′ and *c‐MET* reverse, 5′‐CACCACTGGCAAAGCAAAATAGAAA‐3′ (the GenBank database accession number for these sequences is NC_000007.13); and β‐*actin* forward, 5′‐AGTTGCGTTACACCCTTTCTTG‐3′ and β‐*actin* reverse, 5′‐TCACCTTCACCGTTCCAGTTT‐3′ (the GenBank database accession number for these sequences is NC_000007.14).

Cell culture experiments were carried out in the medical research institute, and RT‐PCR experiments were carried out in the Faculty of Medicine at Banha University.

### Statistical analysis

The results are displayed as the mean ± standard error (SE). One‐way ANOVA was used to analyze the results obtained by Tukey's *post hoc* test. graphpad prism software (version 3.0) (San Diego, CA, USA) was applied to perform statistical analyses. For all statistical tests, the level of significance was set at *P* < 0.05.

## Results

### *In vitro* MTT assay cytotoxicity study

**Fig. 1:**
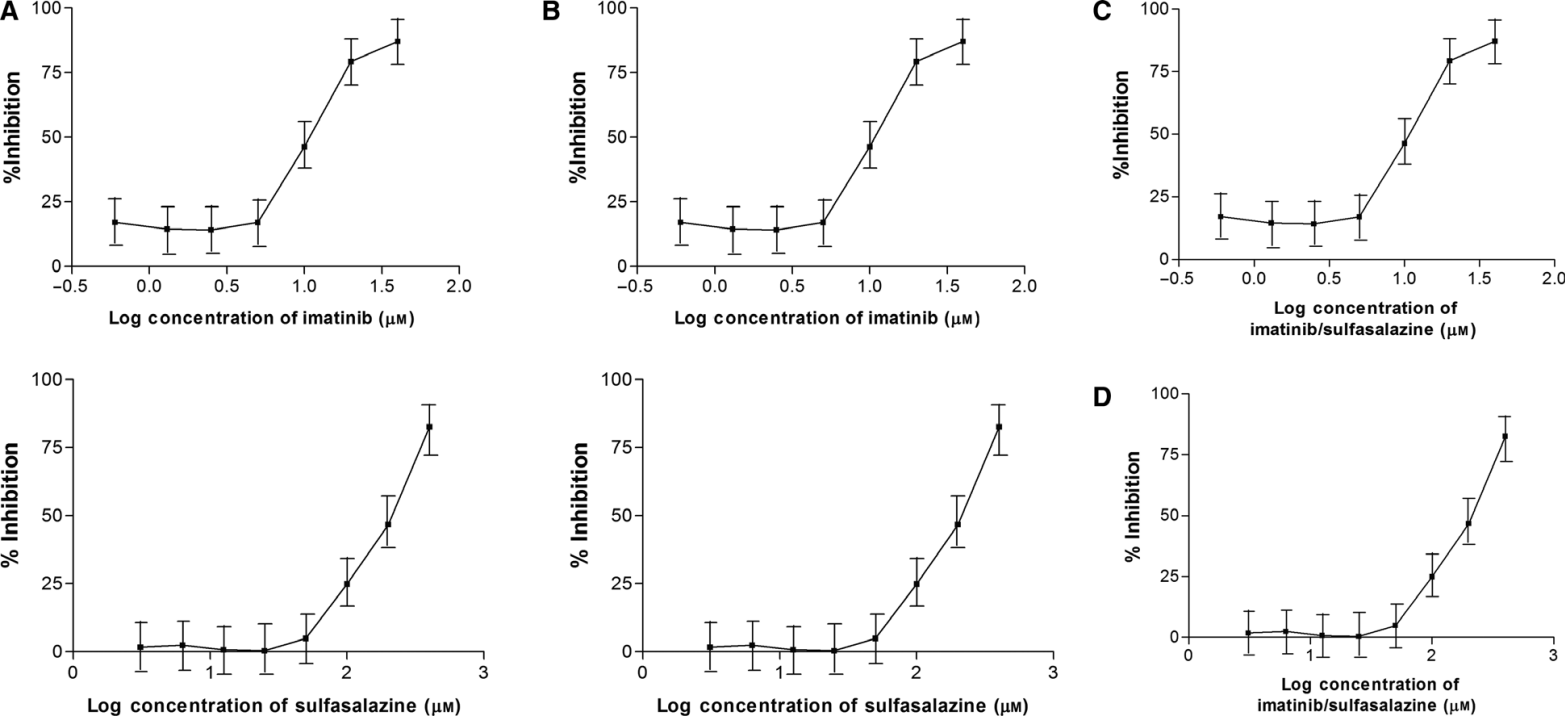
Cytotoxicity results of imatinib and sulfasalazine in (A) HepG2, (B) Huh‐7, (C) combination in HepG2 and (D) Huh‐7 using MTT assay. IC_50_ values were calculated from these curves using graphpad prism 5 software. Each data point was the mean of four independent experiments ± standard deviation.

The half‐maximal inhibitory concentration (IC_50_) values of imatinib were 1.0 and 1.2 μm in Huh‐7 and HEPG2 cells, respectively, whereas the IC_50_ values for sulfasalazine were 250 and 253 μm in the two cell types, respectively. The IC_50_ values for the drugs used in combination were 95 and 100 μm for sulfasalazine and 0.4 and 0.47 μm for imatinib, as shown in Fig. 1. CI calculated using the aforementioned formula indicated that there is a synergistic effect between both medications in which the CI values were 0.78 and 0.79 in Huh‐7 and HEPG2 cells, respectively. These IC_50_ values were found to be equivalent to those previously reported results.

### Effects of imatinib, sulfasalazine and their combination on protein and gene expression levels in Huh‐7 and HEPG2 cell lysates

#### Effects of imatinib (1, 1.2 µm), sulfasalazine (250, 253 µm) and their combination on p‐NFκB, p‐Akt, p‐BCR/ABL, p‐STAT‐3 and caspase‐3 protein levels in Huh‐7 and HEPG2 cell lysates

This work revealed that 3 days of treatment of both cell types with both drugs produced significant decreases in p‐NFκB, p‐Akt, p‐BCR/ABL and p‐STAT‐3 protein levels when compared with the control (*P* < 0.001).

The levels of p‐NFκB, p‐Akt, p‐BCR/ABL and p‐STAT‐3 were significantly decreased in the imatinib‐treated group (25.75%, 59.07%, 61.03% and 28.97%, respectively) in HEPG2 cells and (31.21%, 54.09%, 67.13% and 26.97%, respectively) in Huh‐7 cells compared with the control (*P* < 0.05 and < 0.001).

Furthermore, the administration of sulfasalazine mediated a similar but greater effect on p‐NFκB, p‐Akt, p‐BCR/ABL and p‐STAT‐3 levels in HEPG2 cells (49.83%, 40.93%, 34% and 54.71%, respectively) and in Huh‐7 cells (51.45%, 43.93%, 37.23% and 63.91%, respectively) than in the control (*P* < 0.001).

**Fig. 2:**
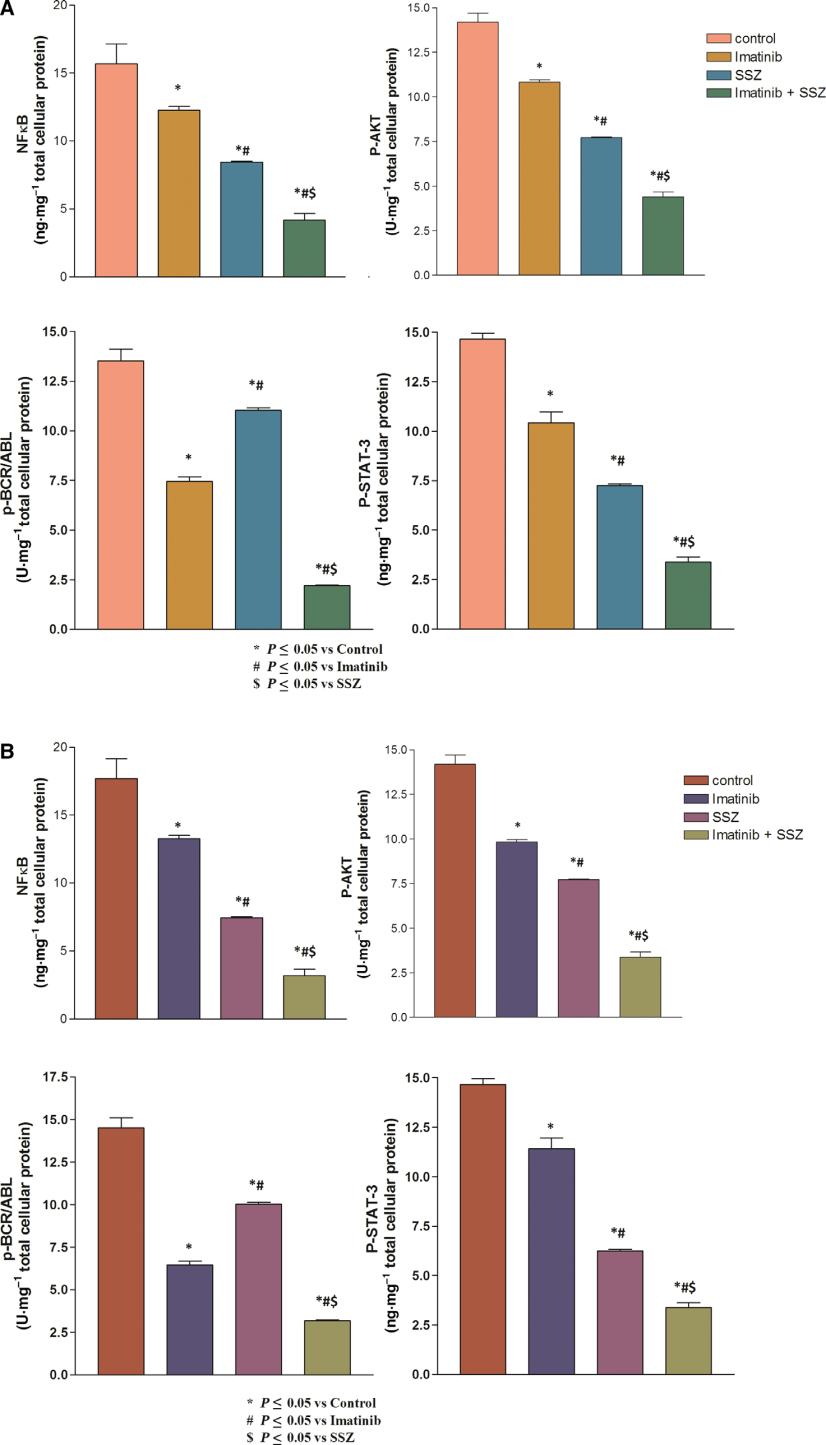
Effects of imatinib (1, 1.2 µm), sulfasalazine (SSZ) (250, 253 µm) and their combination on p‐NFκB, p‐Akt, p‐BCR/ABL, p‐STAT‐3, and caspase‐3 protein levels in (A) HEPG2 and (B) Huh‐7 cell lysates. All treatments were performed for 3 days. The results are presented as the mean ± SE (*n* = 4). Statistical analysis was performed using one‐way ANOVA followed by Tukey's *post hoc* test at *P* < 0.05 compared with the control (*), imatinib‐treated (#) and SSZ‐treated ($) groups.

Impressively, the combination treatment exerted a much more pronounced effect on the aforementioned parameters in HEPG2 cells (95.72%, 79.84%, 87% and 81.93%, respectively) and in Huh‐7 cells (97.12%, 82.14%, 90.1%, and 83.86 %, respectively), an effect that was more significant than either treatment alone, as presented in Fig. 2.

#### **Effects of imatinib (1, 1.2 **µm **), sulfasalazine (250, 253 **µm **) and their combination on *c‐MET* gene expression levels in Huh‐7 and HEPG2 cell lysates**

**Fig. 3:**
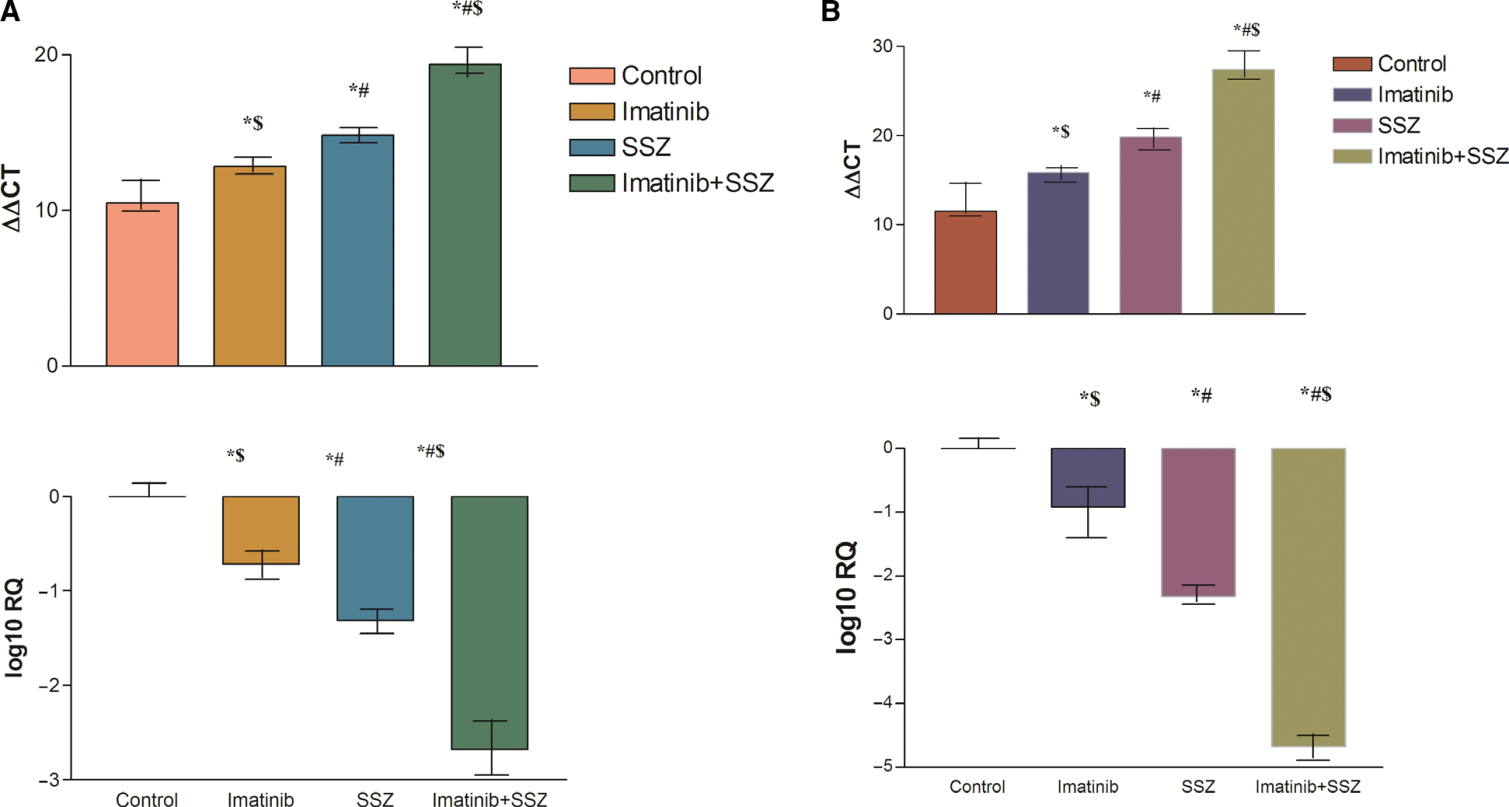
Effects of imatinib (1, 1.2 µm), sulfasalazine (SSZ) (250, 253 µm) and their combination on *c‐MET* gene expression levels in (A) HEPG2 and (B) Huh‐7 cell lysates. All treatments were performed for 3 days. The results are presented as the mean ± SE (*n* = 4). Average fold decrease in *c‐MET* gene expression is shown on the log scale. Statistical analysis was performed using one‐way ANOVA followed by Tukey's *post hoc* test at *P* < 0.05 compared with the control (*), imatinib‐treated (#) and SSZ‐treated ($) groups.

As presented in Fig. 3, imatinib and sulfasalazine caused a marked down‐regulation in the expression of the *c‐MET* gene by 29.64% and 48.74%, respectively, in HEPG2 cells and 31.74% and 49.84%, respectively, in Huh‐7 cells after normalization to the *β‐actin* gene compared with the control.

Interestingly, the combination treatment down‐regulated the expression of the *c‐MET* gene by 89% and 91%, respectively, in the two cell line types in relation to the control, an effect that was significantly different from that of either treatment alone (*P* < 0.001).

#### **Effects of imatinib (1, 1.2 **µm **), sulfasalazine (250, 253 **µm **) and their combination on caspase‐3 and VEGF protein levels in Huh‐7 and HEPG2 cell lysates**

**Fig. 4:**
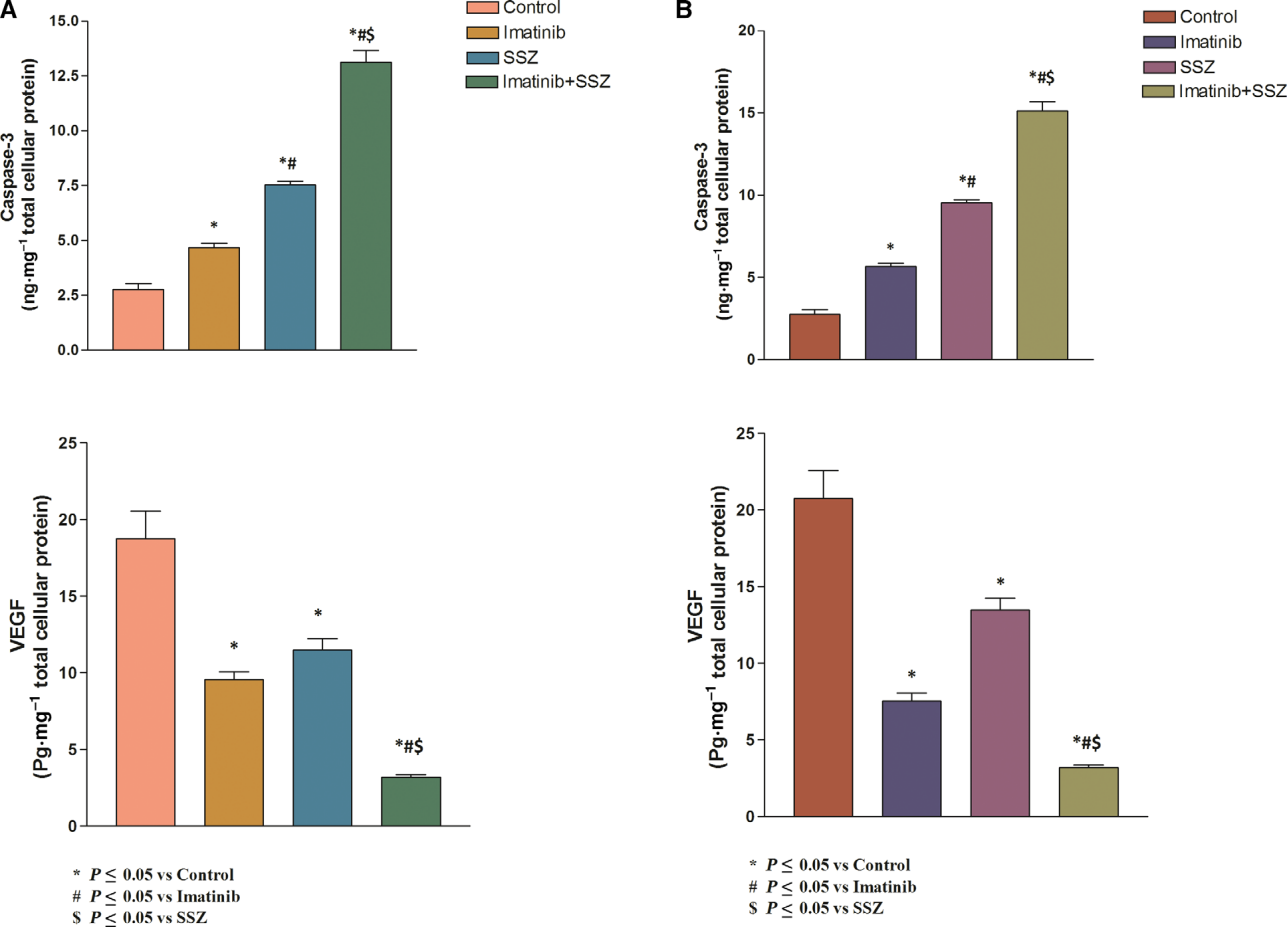
Effects of imatinib (1, 1.2 µm), sulfasalazine (SSZ) (250, 253 µm) and their combination on caspase‐3 and VEGF protein levels in (A) HEPG2 and (B) Huh‐7 cell lysates. All treatments were performed for 3 days. The results are presented as the mean ± SE (*n* = 4). Statistical analysis was performed using one‐way ANOVA followed by Tukey's *post hoc* test at *P* < 0 .05 compared with the control (*), imatinib‐treated (#) and SSZ‐treated ($) groups.

The data in Fig. 4 reveal a significant increase in the level of caspase‐3 among all of the treated groups compared with the control. The imatinib‐treated group demonstrated statistically significant increases in caspase‐3 levels in both types of cell (75.45% and 79.1%, respectively) compared with the control (*P* < 0.05).

Moreover, the administration of sulfasalazine exerted much more pronounced effects on the aforesaid parameter relative to the control (169.82% and 171.92%, respectively), and the increases were statistically significant (*P* < 0.001). Likewise, the combination regimen exerted tremendous effects on caspase‐3 levels (391.09% and 399.1%, respectively) compared with the untreated group. This effect was much better than either treatment alone (*P* < 0.001).

Interestingly, imatinib produced significant decreases in VEGF levels in both types of cells (54.07% and 57.21%, respectively) compared with the control group (*P* < 0.001). Moreover, sulfasalazine produced significant decreases in VEGF protein levels (41.77% and 39.89%, respectively) compared with the control group.

In addition, treatment with a combination of imatinib and sulfasalazine resulted in the highest significant decreases in VEGF protein levels (91.88% and 93%, respectively) compared with treatment with imatinib or sulfasalazine alone.

## Discussion

HCC is considered to be one of the deadliest diseases. Improvements in patient stratification and the development of novel cures have increased patient survival. Collectively, HCC is the sixth most common cause of cancer‐related fatalities worldwide [22, 23]. In recent decades, HCC has been determined to be multifactorial and originate from an enhanced hepatic microenvironment that is related to persistent liver illness and characterized by enormous infection and fibrosis that mediate the deregulation of a few signaling pathways and the collection of hereditary alterations in ordinary hepatocytes [24].

This kind of defect encouraged me to combine sulfasalazine and imatinib to evaluate the effects of both drugs on many of these signaling pathways in HCC. This work is the first to investigate the sites of interaction and the synergism between imatinib and sulfasalazine that could be mediated through the NFκB, BCR/ABL and p‐STAT‐3 signaling pathways. This study demonstrates significant down‐regulation at the *c‐MET* gene level, in which the studies of Santacatterina *et al*. [25] and Mesarwi *et al*. [26] were in concordance with the results that showed that the expression of the *c‐MET* gene was preferentially focused in hepatocytes. Because the *c‐MET* gene was found to be encoding the hepatocyte growth factor receptor, Parizadeh *et al*. [27] pointed out the importance of evaluating the transcriptional level of the *c‐MET* gene.

Moreover, the *c‐MET* gene was found to be a potent proto‐oncogene for hepatocyte growth and regeneration. A previous study demonstrated that *c‐MET* gene expression affects and contributes to the expression of important downstream genes and their pathways, such as NFκB, BCR/ABL and p‐STAT‐3 [28].

In addition, our study revealed that activated p‐NFκB serves as a mediator in HCC by inducing cell viability and growth and causing up‐regulation of antiapoptotic proteins. Our results showed that up‐regulation of caspase‐3 and down‐regulation of Akt and p‐STAT‐3 downstream proteins levels occurred. Up‐regulation of caspase‐3 was found to be mediated through p‐NFκB inhibition.

Our results could be caused by the arguments underlined by Aggarwal [29]: (a) two major inflammation pathways, transcription factors p‐NFκB and p‐STAT‐3, are stimulated by risk factors for cancer; (b) cancer malignancy occurs; and (c) in many cancer types, p‐NFκB and p‐STAT‐3 are continuously active [30].

Our data showed that imatinib produced significant reductions in NFκB, BCR/ABL, p‐STAT‐3, p‐Akt and VEGF protein expression levels, while causing a significant increase in caspase‐3 expression [31]. Our findings corroborate the results of other studies [32, 33, 34, 35, 36, 37, 38], which could be linked to the fact that imatinib causes a reduction in p‐NFκB activity and downstream proinflammatory signaling in lymphocytes. In murine air liquid interface cells, imatinib caused a decrease in lipopolysaccharide‐induced lung p‐NFκB expression.

This work shows the effects of imatinib on the *c‐MET* gene expression level, which could be attributed to the crosstalk between the BCR/ABL and VEGF/VEGFR pathways with collaboration toward boosting propagation and metastasis in endothelial cells [38, 39].

Moreover, this study showed that imatinib caused an increase in caspase‐3 protein expression levels and inhibited VEGF‐independent angiogenesis. Sulfasalazine mediated a comparable pattern with that of imatinib. However, no immediate data correlating sulfasalazine as a potent p‐NFκB and/or VEGF and p‐STAT‐3 inhibitor with HCC exist.

This study revealed for the first time that sulfasalazine can be successful in producing significant reductions in the protein expression levels of p‐NFκB, BCR/ABL, p‐STAT‐3, p‐Akt and VEGF and cause a significant increase in caspase‐3 expression, which is in line with other studies [40, 41, 42, 43, 44, 45], which could be the result of the inhibition of NFκB as one possible pathway for the decrease in VEGF because p‐NFκB is considered to be a potent transcription factor that controls the expression of many downstream proteins, such as BCR/ABL, p‐STAT‐3, p‐Akt and VEGF. In addition, crosstalk between the NFκB and p‐STAT‐3 pathways was found. p‐NFκB was also found to inhibit Akt phosphorylation and BCR/ABL. Another study [46] was in agreement with the present results.

Furthermore, this work revealed for the first time the final result of sulfasalazine on *c‐MET* gene expression in HCC in which previous studies showed that inhibition of *c‐MET* gene expression leads to VEGF protein level inhibition [47]. Our results agree with those from another study [48], which showed that p‐NFκB functions as a prosurvival transcription factor by inducing antiapoptotic genes, including Bcl‐2 family users and caspase‐3 inhibitors. p‐NFκB inhibition was shown to improve apoptosis by accounting for the activation of caspase‐3 in which previous studies prove that inhibition of p‐NFκB leads to repression and inhibition of down‐regulating the BCR/ABL oncoprotein [49].

The sulfasalazine/imatinib combination showed better modulation of AKT/mammalian target of rapamycin signaling and BCR/ABL protein levels. This work shows for the first time the effects of the combination of imatinib/sulfasalazine on p‐NFκB, BCR/ABL, p‐STAT‐3, p‐Akt, VEGF, caspase‐3 and *c‐MET* expression in HCC, thus confirming our aim in this study, which was to assess the potential development of imatinib and sulfasalazine as a combination treatment and to determine whether this enhancement is mediated by these signaling pathways.

Imatinib in combination with sulfasalazine may have the potential as a new treatment option for the cure of advanced HCC. Interestingly, coadministration of sulfasalazine and imatinib resulted in better modulation of the selected molecular pathways by leading to a more significant inhibition of the BCR/ABL, Akt, p‐NFκB and STAT‐3 signaling pathways in addition to a more profound inhibition of inflammation, proliferation and antiapoptotic effects that may be attributed to a chemical interaction between the two drugs when used concurrently.

## Conclusion

Our results based on use of the combination of sulfasalazine and imatinib confirm the synergistic effects that can be derived from the use of an anti‐inflammatory medication with apoptotic, cytotoxic and antiproliferative effects with the tyrosine kinase inhibitor used for the management and cure of HCC. This kind of study should be explored in other types of malignancy and patient with cancer because sulfasalazine can be used as an adjunct remedy to decrease inflammation and spread and can mediate the stimulation of apoptosis in patients with cancer.

## Conflict of interest

The authors declare no conflict of interest.

## Author contributions

Shamaa conceived and designed the study, collected the data, involved in data analysis and interpretation, drafted and critically revised the manuscript, and gave final approval of the version to be published.

## Data Availability

The data that support the findings of this study are available (https://drive.google.com/drive/folders/1m19mMD16jigsd2xDKgroVKbeFq4ZKQ9G?usp=sharing) on request from the corresponding author.
